# PPARs as Key Mediators in the Regulation of Metabolism and Inflammation

**DOI:** 10.3390/ijms23095025

**Published:** 2022-04-30

**Authors:** Manuel Vázquez-Carrera, Walter Wahli

**Affiliations:** 1 Department of Pharmacology, Toxicology and Therapeutic Chemistry, Faculty of Pharmacy and Food Sciences, Institute of Biomedicine (IBUB), University of Barcelona, 08007 Barcelona, Spain; 2 Spanish Biomedical Research Center in Diabetes and Associated Metabolic Diseases (CIBERDEM), Instituto de Salud Carlos III, 28029 Madrid, Spain; 3 Lee Kong Chian School of Medicine, Nanyang Technological University, Singapore 308232, Singapore; 4 Center for Integrative Genomics, University of Lausanne, CH-1015 Lausanne, Switzerland; 5 Toxalim, INRAE UMR 1331, ENVT, INP-Purpan, University of Toulouse, Paul Sabatier University, F-31027 Toulouse, France

Nuclear receptors (NRs) form a large family of ligand-dependent transcription factors that control the expression of a multitude of genes involved in diverse, vital biological processes. Three of these receptors, the peroxisome proliferator-activated receptors (PPARs), were discovered in the 1990s [1,2] and play key roles in the regulation of cellular differentiation, embryonic development, cellular and whole-body metabolism, inflammation, and tumorigenesis in higher organisms. PPARs are activated not only by fatty acids and their derivatives, some of which also signal through membrane receptors, but also by many plant- and marine-derived natural ligands [3]. Furthermore, drugs that target PPARs, such as fibrates and thiazolidinediones, have been developed to treat metabolic diseases. The ‘classic’ molecular mode of action of PPARs in the control of physiological and metabolic processes is via their direct binding, as PPAR:retinoid X receptor (RXR) heterodimers, to peroxisome proliferator response elements (PPREs) in the regulatory regions of target genes (Figure 1). The activity of PPARs can also be modulated by posttranslational modifications and their transcriptional regulatory capacity may present a circadian pattern, depending on their expression and the availability of ligands [3].

Inflammation plays a key role in the progression of metabolic diseases, which has led to numerous studies on the functions of PPARs in the regulation of immune cells and the resolution of inflammation. In fact, all three PPAR isotypes (PPARα, PPARβ/δ, and PPARγ) demonstrate anti-inflammatory capacities. Multiple direct and indirect mechanisms promote the anti-inflammatory effects of PPARs [4]. A much-used mechanism is transrepression, relying on protein–protein interactions, involving different immune cell types (B cells, T cells, macrophages, and dendritic cells). Briefly, the most frequently observed transrepression mechanism is the repression of NF-κB activity through protein–protein interactions between PPARs and the p65 subunit of NF-κB (Figure 1). Other mechanisms are the upregulation by PPAR of IκB, the tethering of the PPARs to activator protein 1 (AP-1), nuclear factor of activated T cells (NFAT), and signal transducers and activators of transcription (STATs), as well as the stabilization of corepressor complexes by ligand-activated PPARs on the promoter of inflammatory genes, which results in their downregulation [4].

At present, there is strong evidence for bidirectional relationships between metabolic and inflammatory processes, with crosstalk between them occurring at different levels, involving all three PPAR isotypes (α, β/δ, and γ) that modulate both metabolic and inflammatory pathways. The strong interest for the multifaceted roles of these receptors in health and disease has led to extraordinary advances in the understanding of metabolism, energy homeostasis, and inflammation. The aim of this Special Issue on “PPARs as Key Mediators of Metabolic and Inflammatory Regulation” was to assemble a broad range of basic and translational original and review articles on the latest discoveries in the regulation of metabolic and/or inflammatory processes by PPARs, in the whole organism of different vertebrate species. Below, we provide a general summary of the topics and data presented in the Special Issue. The scheme in Figure 1 indicates the reference numbers of the articles in the Special Issue, which are grouped according to the topic of their content.

The development of synthetic PPAR ligands and new PPAR binding assays that may help to establish alternative therapeutics with reduced side effects for metabolic and inflammatory diseases has been a promising recurrent activity over the last 25 years. The structural basis of ligand binding to the ligand-binding pocket of PPARs has been explored in much depth to help clarify the molecular mode of action of natural and synthetic ligands, not least through the perspective of drug development. Docking studies by Perez Diaz et al. [5], using computational chemistry methods, revealed that an agonist and antagonist can bind simultaneously to the large ligand-binding pocket of PPARβ/δ without affecting the specificity of one another for the binding domain. Agonist binding followed by simultaneous antagonist binding switches the PPARβ/δ mode of action from induction to repression, as shown by studying the effects of LPS-induced inflammation in the pulmonary artery. In a different approach, Yoshikawa et al. [6] developed coumarin-based PPARγ fluorescence probes for competitive binding assays. Compounds incorporating 7-diethylamino (7-Et2N) coumarin are not difficult to synthesize and can be used in PPARγ binding assays. Such compounds can also be applied in live-cell imaging. Of note, the reported coumarin conjugation strategy could be used to synthesize probes for other nuclear receptors. Honda et al. [7] investigated the PPAR α/βδ/γ selectivity of the clinically approved bezafibrate, fenofibric acid, and pemafibrate, using a cellular transactivation assay, a coactivator recruitment assay, and a thermostability assay. Furthermore, cocrystal structures of the PPARβδ/γ-ligand-binding domains (LBD) and the three fibrates are presented. The results of this study underscore both the differences in the PPAR dual/pan agonistic characteristics of the investigated fibrates and their potential for NAFLD therapy. They also show a way for improved fine-tuning of PPAR isotype selectivity.

The PPARα isotype was first characterized as a member of the receptor family mediating the peroxisome proliferation effect of clofibrate in the rodent liver, hence, its name [1]. It was then found to stimulate peroxisomal and mitochondrial fatty acid β-oxidation pathways (reviewed in [8]). The expression pattern of PPARα in different species and tissues and its functions are discussed by Thari-Joutey et al. [9], who also underscored the important modulation of PPARα activity and function by micronutrients and the possible dietary relevance of these effects. Along a similar line, Hassan et al. [10] summarized the potential of polyunsaturated fatty acids, vitamins, dietary amino acids, and phytochemicals to activate or repress PPARs, describing different mechanisms by which these natural molecules modulate PPARs and how they contribute to prevent metabolic disorders. Special attention is given to transition dairy cows, with insights on how the activity of PPARs could be modulated by nutrigenomic interventions to improve energy homeostasis in dairy animals.

The PPARα isotype is implicated in several metabolic regulations in different organs. In the era of precision medicine, there is a need for highly selective agonists to address treatment gaps, such as the correction of atherogenic dyslipidemia that remains an unmet clinical demand [11]. Using the specific PPARα modulator (SPPARMα) pemafibrate (K-877), which selectively and potently activates PPARα, Lee et al. [12] found that, in addition to stimulating liver function that results in elevated serum levels of fibroblast growth factor 21 (FGF21), a neuroprotective hormone in the eye, PPARα also protects against retinal impairment induced by unilateral common carotid artery occlusion. These observations indicate a possibility of using pemafibrate therapy to improve retinal dysfunction in cardiovascular diseases. Since the first observations in 1996 linking PPARα to the control of inflammation [13], the anti-inflammatory role of this receptor has been very well documented. In their review, Grabacka et al. [14] discussed the interplay of PPARα with different pathways in inflammation, transcription, pattern-recognition receptor signaling, and the endocannabinoid system.

There are several inflammatory skin conditions, such as dermatitis and psoriasis, with the latter being an autoimmune disease. In their study of human psoriatic skin, Sobolev et al. [15] found that PPARγ was downregulated in psoriatic lesions and its expression could be normalized by laser treatment. In this skin condition, PPARγ downregulates the expression of genes promoting the development of psoriatic lesions. The PPARβ/δ isotype also regulates inflammatory pathways, keratinocyte proliferation and differentiation, as well as the oxidative stress response. Its involvement in psoriasis and atopic dermatitis, which is less known, is the focus of the review by Blunder et al. [16], which also debates the relevance of targeting PPARβ/δ to alleviate skin inflammation. One of the major cytokines that drives inflammation is the multifunctional transforming growth factor (TGF)β, which also promotes fibrosis. PPARγ dampens these two processes, highlighting crosstalk between TGFβ and PPARγ, which has been implicated in pulmonary arterial hypertension and kidney failure, in which similar, but also different, mechanisms are involved [17]. The therapeutic potential of the PPARγ agonist pioglitazone against these two conditions is discussed.

PPARs have critical roles in the main functions and homeostasis of metabolic organs, such as the muscle, adipose tissue, and liver. Crossland et al. [18] provided an overview, focusing on the effects of PPARβ/δ agonists on the ability of skeletal muscle to contract to generate force and in the regulation of the necessary metabolic support, highlighting observations from in vivo/ex vivo animal models and human volunteers. They also focus on the potential role of PPARγ in reducing muscle inflammation and the metabolic disorders caused by sepsis. Interestingly, synthetic ligands of PPARβ/δ can enhance performance in athletes and are included as S4.5 Metabolic Modulators in the World Anti-Doping Agency’s (WADA) Prohibited List. Sibille et al. [19] investigated whether a specific signature in blood T cells could identify the ingestion of the prohibited PPARβ/δ agonist GW0742. PPARβ/δ activation by GW0742 has been shown to stimulate fatty acid oxidation (FAO) in mouse and human T cells, with increased Treg polarization of human primary T cells. Interestingly, PPARβ/δ activation increases FAO in mouse blood T cells too, but this effect is obscured by training, indicating that this signature cannot be used to control doping.

Adipose tissue is a key organ for maintaining healthy energy homeostasis and its dysfunction is often associated with pro-inflammatory, hyperlipidemic, and insulin-resistant environments that promote type 2 diabetes and metabolic syndrome. Sun et al. [20] summarized the roles of the three PPAR isotypes in the metabolic processes and differentiation of white, beige, and brown adipocytes and how they contribute to maintaining metabolic homeostasis in fat.

Non-alcoholic fatty liver disease (NAFLD) is a major health issue all around the world and is often associated with type 2 diabetes and obesity. Initial steatosis, identified by lipid accumulation in hepatocytes, can progress to non-alcoholic steatohepatitis (NASH), which is characterized by inflammation and various levels of fibrosis and is associated with an increased risk of cirrhosis and hepatocellular carcinoma. PPARs regulate many processes that are impaired in NAFLD, such as lipid and glucose metabolism, as well as inflammation. Therefore, PPARs have emerged as attractive clinical targets for NAFLD [21].

In their review, Monroy-Ramirez et al. [22] provided updated information on the critical roles of PPARs in the mechanisms involved in the genesis of several liver diseases and how these receptors could be engaged in therapeutic scenarios. Using a mouse model of metabolic syndrome with an altered expression of PPARα and γ, Cano-Martinez et al. [23] studied the mechanisms underlying the beneficial effects of the polyphenols resveratrol (RSV) and quercetin (QRC) on inflammation in damaged livers. They found a downregulation in the expression of the purinergic receptor P2Y2, neutrophil elastase (NE), and toll-like receptor 4 (TLR4). The repression of these pathways decreased apoptosis and hepatic fibrosis. In the cluster of conditions belonging to metabolic syndrome, type 2 diabetes is a condition with an unmet need for additional treatment options to better control the disease in many patients. PPARβ/δ shows promise, with most of its antidiabetic effects mediated through the activation of AMP-activated protein kinase (AMPK). The review from Aguilar-Recarte et al. [24] outlines the most recent findings on the PPARβ/δ-AMPK antidiabetic pathway, consisting of the upregulation of glucose uptake, fatty acid oxidation, and autophagy, as well as muscle remodeling and the inhibition of endoplasmic reticulum stress and inflammation. Understanding the mechanisms underlying the activation of the PPARβ/δ-AMPK pathway may result in the development of new therapies to prevent and treat the disease and insulin resistance. Along a similar line of thought, Lange et al. [25] provided a general overview of current PPAR-targeting treatments of NAFLD and NASH in patients with type 2 diabetes. This important knowledge on treatments was gained from both clinical trials and observational studies. Lange et al. also considered treatment outcomes on obesity, dyslipidemia, and cardiovascular disease that are often associated with NAFLD/NASH and discussed agonists currently in clinical trials. Finally, sexually dimorphic effects of PPAR-targeting interventions are addressed. There is indeed mounting evidence for sexual dimorphism in NAFLD, with men being more affected than women. Shiffrin et al. [26] studied sexual dimorphism in different mouse models, including those with PPARγ deletion. They found a clear sexual dimorphism in lipodystrophic fat-specific *Pparg*-null mice. The mutant females developed macro- and microvesicular hepatosteatosis, which was lost in gonadectomized mutant mice. In all the tested models, hepatosteatosis strongly impacted sex-biased gene expression in the liver.

Currently, increasing attention is being given to the PPAR epigenetic landscape, as presented by Porcuna et al. [27]. This landscape comprises epigenetic effectors, PPAR regulators, and PPAR-regulated factors, including epigenetic enzymes, DNA methyltransferases, histone modifiers, and non-coding RNAs. The focus of the review of Porcuna et al. is on PPARα- and PPARγ-related epigenetic regulation in obesity, diabetes, immune disorders, and cancers. The possible therapeutic use of PPAR-controlled epigenetic modulation is also discussed.

Gold [28] summarized the roles of PPARγ in depression, the second largest cause of disability worldwide, according to the World Health Organization. Genetic predisposition alongside recurrent social and other stressors reduce neuronal resilience that can result in the development of depression. In this condition, extreme endoplasmic reticulum stress responses, glutamate toxicity, parainflammation, brain-derived neurotrophic factor (BDNF) function, and the down-regulation of central and peripheral insulin signaling are enhanced. As detailed in the review, the PPARγ system can modulate and dampen all these pathological mechanisms. It is proposed that PPARγ agonists may have significant antidepressant effects, which remain to be explored further.

PPARs are involved in mycobacterial and viral infections. Grabacka et al. [14] summarized PPARα-specific immunomodulatory functions during infections by parasites, bacteria, and viruses, as well as the modulation of processes associated with innate immunity. Tanigawa et al. [29] discussed the advancement in understanding PPARs in host–mycobacteria crosstalk via their impact on the host-dependent mechanisms of lipid metabolism, anti-inflammatory processes, and autophagy during infection. PPARγ is activated in macrophages infected with Mycobacterium leprae or Mycobacterium tuberculosis and regulates some genes involved in the uptake and accumulation of lipids and in cellular metabolism. Mycobacteria use the triacylglycerol (TAG) and cholesterol derived from the host as nutrients and support for evading the host immune system. Layrolle et al. [30] provided an update of the little-known role of PPARγ in viral infections of the brain parenchyma. Viruses can overcome the defensive pathways of host cells to replicate and spread. In these processes, PPARγ becomes a critical target. There is strong evidence for its involvement in brain or neural cells infected by human immunodeficiency virus 1 (HIV-1), the Zika virus, and cytomegalovirus. In fact, PPARγ is a double-edged sword with respect to the triad of neurogenesis, viral replication, and inflammation. In an infected adult brain, PPARγ is beneficial against inflammation, oxidative stress, and viral replication. In this context, PPARγ agonists are considered to be candidate drugs in the treatment of HIV-1-induced brain inflammation to improve neurocognitive outcomes. On the contrary, PPARγ activation is deleterious in neurogenesis in a developing brain, as observed in human cytomegalovirus infections and possibly in Zika viral infections as well. Notably, the activation of PPARγ during an infection of developing brains by human cytomegalovirus promotes viral replication.

In conclusion, the amazing pleiotropy of the three PPAR isotypes, with such diverse effects on different processes and organs, is highlighted once more herein. Future PPAR research, which is needed more than ever, will undoubtedly uncover many other roles of these ligand-activated transcription factors in all vital metabolic and physiological pathways. Further exploration of the vast ensemble of natural ligands, many probably still to be identified, is also needed, which will uncover more about how PPARs contribute to the adaptation of organisms to their environment, in terms of nutrition, toxic substances, infectious agents, and strong temperature fluctuations, just to mention a few of the environmental agents and factors. The use of potent specific synthetic ligands interacting with one or more PPAR isotypes simultaneously offers a very broad avenue for advances in precision medicine.

## Figures and Tables

**Figure 1:**
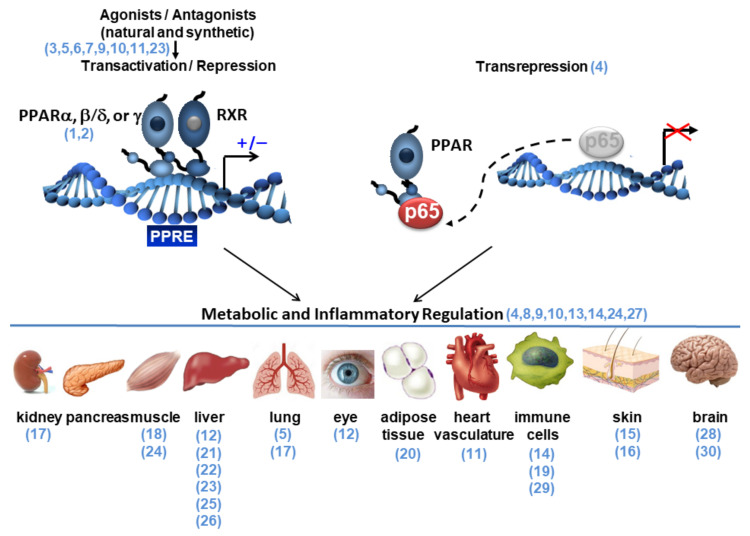
Modes of action of PPARs in transcription transactivation and transrepression (only one of the several mechanisms of transrepression is shown). A non-exhaustive list of the target organs subjected to PPAR-mediated metabolic and inflammatory regulation is shown. The reference numbers (in blue and in parentheses) refer to the articles quoted in this Special Issue (see References); in the figure, they are listed and grouped according to the topic addressed in the corresponding articles. PPRE: peroxisome proliferator hormone response element. p65 subunit of NF-κB. RXR: retinoid X receptor.
